# The Pivotal Role of Preclinical Animal Models in Anti-Cancer Drug Discovery and Personalized Cancer Therapy Strategies

**DOI:** 10.3390/ph17081048

**Published:** 2024-08-09

**Authors:** Haochuan Guo, Xinru Xu, Jiaxi Zhang, Yajing Du, Xinbing Yang, Zhiheng He, Linjie Zhao, Tingming Liang, Li Guo

**Affiliations:** 1 Jiangsu Key Laboratory for Molecular and Medical Biotechnology, School of Life Science, Nanjing Normal University, Nanjing 210023, China; 231212007@njnu.edu.cn (H.G.); 211202068@njnu.edu.cn (X.X.); 231202046@njnu.edu.cn (J.Z.); 221202100@njnu.edu.cn (Y.D.); 221202124@njnu.edu.cn (X.Y.); 2 State Key Laboratory of Organic Electronics and Information Displays & Institute of Advanced Materials (IAM), Nanjing University of Posts & Telecommunications, 9 Wenyuan Road, Nanjing 210023, China; 1023173006@njupt.edu.cn (Z.H.); 1223014120@njupt.edu.cn (L.Z.)

**Keywords:** preclinical animal models, anti-cancer therapy, drug development, personalized therapy

## Abstract

The establishment and utilization of preclinical animal models constitute a pivotal aspect across all facets of cancer research, indispensably contributing to the comprehension of disease initiation and progression mechanisms, as well as facilitating the development of innovative anti-cancer therapeutic approaches. These models have emerged as crucial bridges between basic and clinical research, offering multifaceted support to clinical investigations. This study initially focuses on the importance and benefits of establishing preclinical animal models, discussing the different types of preclinical animal models and recent advancements in cancer research. It then delves into cancer treatment, studying the characteristics of different stages of tumor development and the development of anti-cancer drugs. By integrating tumor hallmarks and preclinical research, we elaborate on the path of anti-cancer drug development and provide guidance on personalized cancer therapy strategies, including synthetic lethality approaches and novel drugs widely adopted in the field. Ultimately, we summarize a strategic framework for selecting preclinical safety experiments, tailored to experimental modalities and preclinical animal species, and present an outlook on the prospects and challenges associated with preclinical animal models. These models undoubtedly offer new avenues for cancer research, encompassing drug development and personalized anti-cancer protocols. Nevertheless, the road ahead continues to be lengthy and fraught with obstacles. Hence, we encourage researchers to persist in harnessing advanced technologies to refine preclinical animal models, thereby empowering these emerging paradigms to positively impact cancer patient outcomes.

## 1. Introduction

Annually, millions of individuals worldwide are diagnosed with cancer, posing a formidable challenge to public health owing to the disease’s diverse nature, ever-evolving characteristics, and propensity for drug resistance [1]. The multifaceted mechanisms underlying cancer initiation, progression, and metastasis require comprehensive study given their involvement in multiple systemic abnormalities and pathways [2]. To unravel the interplay and complexities inherent in cancer, scientists have increasingly harnessed preclinical animal models as a pivotal bridge spanning the divide between laboratory investigations and clinical practice. However, the development of effective and safe anti-cancer therapies remains hindered by numerous obstacles, including the lack of models accurately replicating human disease, the substantial failure rate of drug candidates during clinical trials, and the ethical quandaries surrounding research involving human subjects. These challenges underscore the paramount importance of refining preclinical animal models and deepening our comprehension of their role in the discovery and development of cancer therapeutics.

The research and development of novel drugs exert a profound influence on the medical industry, presenting substantial opportunities. Recent milestones, exemplified by the Food and Drug Administration (FDA) approval of Amgen’s Kirsten rat sarcoma viral oncogene-G12C (KRAS-G12C) inhibitor sotorasib [3,4,5], have provided valuable insights into cancer treatment. In addition to submitting an application to the FDA [6], preclinical and clinical studies are typically conducted during the new-drug development process [7,8,9].

Before progressing to human trials, drug candidates must undergo a rigorous and comprehensive evaluation process to assess their potential for acute, chronic, developmental, and reproductive toxicity and carcinogenicity. This exhaustive examination is imperative to guarantee the efficacy and safety of the drugs post-administration, ensuring that they meet the highest standards of patient care and well-being [10,11]. Preclinical studies focusing on clinical pharmacology [12], pharmacokinetics [13], and toxicology are conducted in small animals or at the cellular and molecular levels to predict potential effects of chemicals on humans [14]. Furthermore, preclinical studies can also determine the maximum tolerable dose for human clinical trials in order to minimize risks [15]. The establishment of preclinical animal models offers researchers with a multi-dimensional understanding of cancer development. These models provide a more comprehensive insight into cancer characteristics than traditional cell culture methods [16]. Selecting appropriate animal models not only maximizes disease replication but also eliminates ethical concerns associated with human experiments [17]. These models have proven crucial in various aspects, including pinpointing effective sites for cancer therapy [18], facilitating the real-time analysis of signaling pathways during tumor treatment [19], enhancing early cancer detection and prevention strategies [20], assessing the toxicity of biochemical agents [21], and advancing the clinical application and development of novel drugs. This paper provides a detailed description of the application of preclinical animal models in cancer therapy, covering the establishment and classification of models, recent research progress, development of new drugs based on biological characteristics at different stages of tumor development, guidance for personalized anti-cancer therapy, and targeted selection in safety evaluation experiments. The objective of this review is to facilitate researchers in the selection of suitable preclinical animal models for experiments and to promote the enhancement and development of optimized preclinical animal models while adhering to pertinent laws, regulations, and ethical principles regarding animals.

## 2. Classification and Establishment of Preclinical Animal Models and Recent Progress

**Figure 1:**
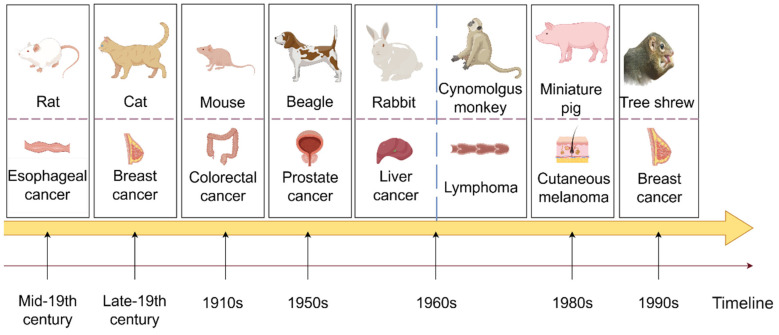
A comprehensive illustration of the developmental timeline, showcasing the progression of various preclinical experimental animal models over time (By FigDraw).

The evolution of preclinical animal models boasts a rich and extensive historical tapestry. Beyond the ubiquitous mouse model, a diverse array of animals, including cats and rabbits, have played pivotal roles in the early stages of animal experimentation. A comprehensive illustration of the developmental timeline, showcasing the progression of various preclinical experimental animal models across time (Figure 1), will be described in subsequent sections. In this section, we separate preclinical animal models into distinct categories.

### 2.1. Rodentia (Mainly Mice)

Rodentia, primarily comprising mice, rats, and guinea pigs, are at the forefront of preclinical animal models, particularly in the field of drug development. Their usefulness stems from a range of advantages, including genetic manipulability, cost-effectiveness, and physiological similarities to humans that facilitate the replication of complex disease states. In the pharmaceutical pipeline, rodent models have accelerated the understanding of disease mechanisms and expedited the discovery and validation of new therapeutics.

Mice serve as a prime example and have experienced a revolutionary expansion in their applications, transitioning from traditional transplantation models to advanced platforms incorporating in vivo gene editing techniques and orthotopic tumor modeling. This evolution has paralleled advancements in biotechnology that enable the precise manipulation of the murine genome to more accurately mimic human disease conditions than ever before. The timeline of this progression highlights key milestones such as the introduction of transgenic mice in the 1990s followed by conditional gene targeting and Clustered Regularly Interspaced Short Palindromic Repeats/CRISPR-associated protein 9 (CRISPR/Cas9)-mediated genome editing in subsequent decades.

These technical advances have propelled rodent models into central roles in several areas of cancer research. They have been invaluable for identifying therapeutic targets, enabling the real-time monitoring of signaling pathways during treatment, and enhancing early detection and intervention strategies. Additionally, they provide crucial platforms for toxicity assessments of bioactive compounds and the preclinical evaluation of emerging anti-cancer drugs.

Despite their prominence, rodent models do present certain limitations. Genetic and physiological differences with humans can lead to discrepancies in drug responses and disease manifestations. Additionally, the oversimplified immune microenvironment in some models may not fully recapitulate the complexity of human immunity, potentially affecting the translation of immunotherapy findings. Furthermore, while orthotopic and genetically engineered models offer improved realism, they can be technically challenging and resource-intensive.

Another significant concern that cannot be overlooked is the ethical consideration regarding animals. Mice are the most commonly utilized animals in preclinical research, and their numbers used are undeniably substantial. However, there remains a notable deficiency in welfare ethics within large-scale experiments. For instance, mice often experience substandard experimental environments and inadequate nutritional supplementation. It is well-known that environmental temperature, humidity, light, noise, odor, and changes in feed nutrition significantly impact the health and welfare of experimental animals due to their high sensitivity. Improper feeding and management practices as well as rough handling during transportation can lead to restlessness, panic, pain, and injury among mice. The fundamental principle of animal experimentation ethics lies in the “3R” principle: replacement, reduction, and refinement. Despite its global acceptance as a guiding principle for animal experiments worldwide, awareness of this principle remains generally low among domestic researchers. Furthermore, instances of mistreatment or abuse by experimenters towards mice occur from time to time during mouse experiments; incorrect or inhumane methods of euthanasia are also observed, along with the indiscriminate disposal of animal carcasses. Therefore, regardless of which mouse model is employed, it is imperative to establish a foundational ethic centered on treating them with reverence while upholding a sense of responsibility and compassion.

**Figure 2:**
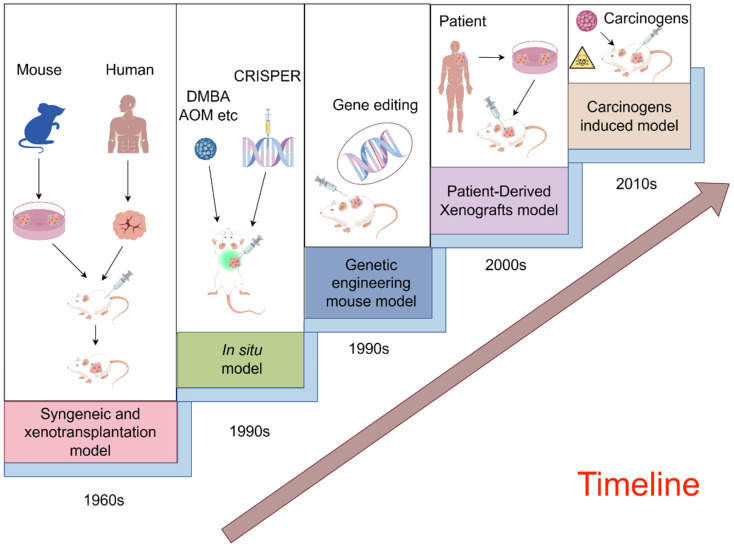
A schematic illustration of the evolution of preclinical mouse animal models for cancer therapy (By FigDraw).

With the progress of technology and theory, preclinical animal models continue to break through their limitations and evolve into more diverse types. In the case of mice, applications are expanding from simple transplantation models to complex in vivo gene editing and orthotopic modeling [22,23]. A schematic illustration of the evolution of preclinical mouse animal models for cancer therapy is provided in Figure 2 [24,25,26,27,28,29,30]. To illustrate, we introduce and summarize five prevalently employed mouse models, underscoring their significance in the field.

#### 2.1.1. Syngeneic and Xenotransplantation Models (Subcutaneous, Intraperitoneal, Intravenous, and Intramuscular)

According to the source of the graft, it can be categorized into a syngeneic transplantation model and xenograft model. Syngeneic transplantation models involve using immunodeficient mice as recipients for the regrowth of spontaneously generated tumor tissue or an ex vivo cultured tumor cell line [31,32]. In xenograft models, immunodeficient mice are used as recipients for tumor growth after inoculation with a different human tumor line or primary tumor tissue from a patient [33,34]. Transplantation models have been utilized to directly induce tumor formation in mice with carcinogens and evaluate the anti-tumor effect of tumor immunotherapy in these tumor-bearing mice [35,36].

Different transplantation models have their own advantages and disadvantages. Syngeneic transplantation has the advantage of consistent tissue source [37], while xenotransplantation can prepare various animal models including human tumors and more accurately construct the original tumor microenvironment [38]. Homologous tumor mouse models, however, may not be capable of fully representing the complexity of human tumor clinical cases and exhibit immune phenotypic differences [39]. Besides, there is a species mismatch between the tumor and the host microenvironment in the xenograft model [40].

In addition, the range of species available for syngeneic transplantation models is limited. Even if a homologous model platform has more than 30 models, it still falls short compared to the variety provided by human xenotransplantation models. Furthermore, the performance in specific cancer types (such as lung cancer) is inadequate and not universally available across all model types [41]. Lastly, despite the relative stability of the model, results from each study may vary. The reasons for these differences are yet to be determined and could possibly be attributed to factors such as microbiome, location, or feeding style of the mice; assessing how these factors might impact the checkpoint inhibitor response is essential [42].

The xenotransplantation model is no exception. Traditional tumor xenotransplantation models, using in vitro cell lines, present a microenvironment that differs significantly from primary tumors due to the absence of tumor-related matrix and blood supply. The genetic variation and tumor heterogeneity introduced by these cell lines make it difficult to predict drug effects in clinical trials accurately. With an average success rate of less than 8% for translating results from animal tumor-bearing models to clinical applications, it is evident that this model fails to faithfully reflect the carcinogenic process in humans [43]. Highly anaplastic cancer cells cultured in vitro represent extreme derivatives of highly advanced cancer, independent of the primary tumor matrix—a key factor in tumor metastasis [44]. Furthermore, critical genetic, molecular, immune, and cellular differences between humans and mice have hindered this model’s effectiveness as a means of personalized cancer therapy [45,46].

In a syngeneic mouse model, Olivier et al. devised a therapy employing AMG509 (also referred to as xaluritamig) to target metastatic castration-resistant prostate cancer (mCRPC). The findings demonstrated that AMG 509 effectively elicited T-cell-dependent cytotoxicity in prostate cancer cell lines in vitro and facilitated tumor regression in allograft mouse models [47]. In the xenograft model, a Non-small cell lung cancer (NSCLC) mouse model was used to study the effect of aerobic exercise on radiotherapy efficacy [48].

#### 2.1.2. In Situ Model

The orthotopic tumor model involves the induction of tumor formation in mice at the original site, providing a more realistic simulation of tumor occurrence and development in the human body compared to the classical transplanted tumor model [49]. The main methods include chemical carcinogen induction and gene-editing technology induction [50]. Chemical carcinogenesis selectively induces malignant tumors in specific organs or tissues of mice using carcinogens, while gene-editing induction utilizes tools such as CRISPR to induce the development of specific tumor types in mice through targeted knockout or the activation of oncogenes or tumor suppressor genes [51].

In situ models have a broad spectrum of applications. Compared to the xenograft tumor models mentioned above, the in situ tumor model closely mimics the natural environment of the original tumor, encompassing complete pathological and immune responses. The establishment of stable, reliable, and reproducible in situ animal models is crucial as it offers an opportunity to investigate pathogenesis and malignant progression, such as local invasion and the distal metastatic spread of the primary tumor. This is essential for the discovery and development of new therapeutic agents. In situ implantation combined with the subsequent harvesting of metastatic sites can generate cancer cell variants associated with clinical metastasis processes. Consequently, in situ models can more accurately replicate the organ microenvironment that determines tumor cell phenotypes, including interstitial microenvironment interactions’ role in tumor growth and metastasis compared to heterotopic tumor xenotransplantation models [52]. They also enable the evaluation of the impact of targeted drugs or cytotoxic drugs on tumor growth within the relevant microenvironment. Additionally, they facilitate the assessment of local tumor invasion, metastasis, and resistance, as well as preclinical survival endpoints associated with continuous or intermittent treatment using novel drug dose combinations [35,36]. The in situ model is also widely utilized by researchers. Taking the above two methods as examples, the chemical carcinogen induction method is characterized by its ease of manufacture and high success rate, with commonly used substances such as 7,12-Dimethylbenz[a]anthracene (DMBA) and Azoxymethane (AOM) [53,54,55,56]. Gene editing technology can elucidate the molecular mechanism of carcinogenesis and simulate the occurrence process, such as CRISPR.

However, the in situ model also presents clear disadvantages. The induction of chemical carcinogens has a toxic effect on normal cells, and gene-editing technologies are not only costly and complex but also have limited success rates.

Researchers have made significant advancements in orthotopic models. Yang utilized the Azoxymethane/Dextran sodium sulfate (AOM/DSS) mouse model to investigate the role of Mex-3 RNA Binding Family Member A (MEX3A) in colorectal cancer [57], while Clements employed piggyBac transposition and CRISPR-Cas9-mediated somatic glioblastoma mouse models for further exploration [58].

#### 2.1.3. Genetically Engineered Mouse Model

Genetically engineered mouse models are created by manipulating key genes related to human tumorigenesis at the level of the mouse genome. Commonly used models include:

(1) Gene knockout mice: specific tumor suppressor genes are knocked out or oncogenes are overexpressed to induce spontaneous tumor formation, allowing for the systematic evaluation of oncogene and tumor suppressor function.
(2) Humanized mouse model: mice are genetically modified to mimic human tumors by introducing human oncogenes or transplanting human tumor tissues, faithfully replicating the human tumor microenvironment and widely used for in vivo drug evaluation [59].

Genetic engineering models can be utilized to validate potential tumor genes and drug targets, assess therapeutic efficacy, analyze the impact of the tumor microenvironment, and evaluate mechanisms of drug resistance [35,36]. Despite the technical challenges posed by factors like cost, lengthy production cycles, and limited representativeness, genetic engineering models continue to enjoy widespread popularity among researchers owing to their unparalleled capacity to precisely mimic genetic alterations in cancer cells. In fact, numerous cancer treatment strategies rely heavily on genetic engineering mouse models, underscoring their indispensable role in advancing our understanding and treatment of cancer. Revskij et al. used the Uncoupling Protein 2 knockout (Ucp2 KO) mouse model to explore the effect of Ucp2 on the microenvironment of pancreatic ductal adenocarcinoma (PDAC) [60], while Han et al. focused on the application of humanized immune system (HIS) mouse model in Chimeric Antigen Receptor T-Cell Immunotherapy (CAR-T) therapy [61].

#### 2.1.4. Patient-Derived Xenograft Model (PDX)

The PDX model involves the direct transplantation of a fresh biopsy sample from a patient tumor into immunodeficient mice to establish animal models [62]. In contrast to the traditional cell-derived xenografts model, PDX can maintain the genetic and epigenetic diversity of tumors in vivo, reproducing characteristics such as growth rate, drug sensitivity, and drug resistance observed in humans [63].

PDX models are increasingly favored for their clinical relevance and predictive value, providing insights into treatment efficacy for personalized medicine. They also serve as a valuable platform for drug screening and development, as well as for advancing our understanding of tumor biology [35,36,64]. Huang’s team utilized NOD-SCID-IL2RgammaC-null mice to establish a PDX model using tumor tissue from patients with AT-Rich Interaction Domain 1A (ARID1A)-mutated lung cancer [65]. Dankner employed PDX mouse models of highly invasive and minimally invasive brain metastasis to further investigate the impact of reactive astrocyte-specific signal transducer and the activator of the transcription 3 (STAT3) gene on minimally invasive brain metastasis growth in vivo [66]. The primary limitation of PDX models stems from their dependency on surgical resection as the primary acquisition route for tumor tissue, a process that is challenging to repeat frequently and characterized by an inconsistent success rate.

#### 2.1.5. Carcinogen-Induced Models

Chemical carcinogenesis provides a straightforward approach to establish an orthotopic carcinoma model in mice. Various types of tumors can be induced in mice by researchers using exogenous carcinogens. Currently, commonly used carcinogens include pyrene compounds [67], heterocyclic aromatic amines [68], nitrosoamine compounds, and others [69]. These substances can induce skin cancer, liver cancer, lung cancer, breast cancer, and other solid tumors in mice through different exposure pathways. This model closely resembles the molecular, biochemical, and histopathological characteristics of specific human cancers, such as hyperplasia, dysplasia, malignant prelesions, low-grade highly differentiated carcinomas, and eventually invasive and poorly differentiated carcinomas capable of varying degrees of metastasis to the local and distal organ sites of the host [35,36]. In various fields, we often see the use of chemical carcinogenic models. For example, Sotty et al. induced liver cancer in mice with diethylnitrosamine (DEN); DEN is a potent chemical carcinogen capable of inducing numerous cancer-causing mutations following a single injection. The primed hepatocytes are subsequently cloned and amplified in a proliferative environment, rendering the DEN model a robust carcinogenic agent. In rodent studies, DEN has been widely utilized in experimental liver cancer research, mimicking various aspects of human hepatocellular carcinoma (HCC), including angiogenesis, metabolic reprogramming, immune dysfunction, and metastatic potential [70]. While Nithya et al. induced lung cancer in Swiss albino mice with benzopyrene, benzo[a]pyrene is a potent carcinogen for both humans and animals. Initially identified as a cause of skin cancer, further research has revealed its carcinogenic effects on various organs, including the lungs, liver, esophagus, and gastrointestinal tract. In an experimental study involving mice with induced lung cancer from benzopyrene exposure, elevated levels of lipid peroxides were observed along with decreased antioxidant status and histological aberrations [67].

There are differing opinions about the chemical carcinogenic model. The advantages include its simplicity of operation, low time cost, and good intervention before the occurrence of cancer; however, there are also several shortcomings, such as unclear mechanisms of carcinogenesis which affects result accuracy due to toxicity from the carcinogens themselves, leading to a gap between experimental differentiated tumors and human tumors. As previously mentioned, both diethylnitrosamine and benzopyrene are carcinogenic and highly toxic. Therefore, special precautions should be taken when using carcinogenic compounds in preclinical animal experiments. Researchers must implement specific protective measures, and experimental animals should receive specialized treatment to prevent leakage and potential human infection upon euthanasia. Lastly, the impact of carcinogens on preclinical animal experiments represents a significant limitation that requires careful consideration. Table 1 provides a comprehensive summary of the strengths and weaknesses of five distinct mouse models, along with highlighting their advancements in the context of specific cancer types.

### 2.2. Large Animals (Lagomorphs, Suids, Scandentia, Carnivores, Primates, etc.)

The utilization of large animals in preclinical experiments holds significant value. Firstly, in comparison to small animals such as mice and rats, large animals like dogs, pigs, and rabbits exhibit greater physiological similarity to humans in terms of metabolic characteristics and disease occurrence. Consequently, medical experiments involving large animals can more accurately replicate the onset and progression of human diseases, thereby offering robust support for the study of human ailments. Secondly, within the realm of new drug research and development, experiments conducted on large animals represent a pivotal stage for assessing drug safety. By observing drug efficacy, side effects, pharmacokinetics, and other attributes in large animals, crucial evidence for drug clinical trials can be obtained while simultaneously mitigating the risks associated with such trials.

However, large animals also pose significant limitations in preclinical experiments. On the one hand, the operational complexity of conducting experiments on large animals demands a high level of skill and experience from researchers. Precise control is essential for surgical procedures, drug administration, data collection, and other processes to ensure the accuracy of experimental results. On the other hand, ethical considerations are paramount when conducting experiments involving big animals; a strict ethical review is necessary due to potential welfare concerns, such as pain, disability, and even mortality.

Thus, the challenge lies in striking a balance between its value and complexity, which is a crucial concern in the field of biomedical research. This section delves into several typical aspects preceding clinical experiments in large animals, offering researchers fresh insights and methodologies by drawing parallels with human biological characteristics and advancements in cancer therapy.

#### 2.2.1. Lagomorphs (Mainly Rabbit) 

In the field of large animal models for cancer drug development, rabbits have attracted attention due to their suitability for translational research. Rabbits (*Oryctolagus cuniculus*) are mammals belonging to the order Lagomorpha. The genus Oryctolagus is primarily used for experimental purposes, while hares (*Lepus*) and white-tailed brown rabbits (*Sylvilagus*) are also included. Their ease of handling, non-aggressive behavior, cost-effectiveness compared to larger mammals, rapid reproduction, and wide availability make them a practical alternative for preclinical testing [71]. A notable example of their utility lies in the innovative work conducted by Li et al., who utilized oxaliplatin to develop a temperature-sensitive liquid embolization agent, thereby effectively demonstrating a targeted therapy for VX2 gastric cancer in rabbits [72]. This approach exemplifies how rabbits can facilitate the advancement and validation of novel anti-cancer therapeutics, highlighting their importance in the complex process of cancer drug development.

#### 2.2.2. Suids (Mainly Miniature Pigs)

The anatomical, physiological, genetic, and immunologic similarities between humans and pigs make pigs an integrated preclinical model of human disease [73]. Miniature pigs are utilized in biomedical research as subjects. They are classified under the phylum Mammalia, the order Artiodactyla, the family Suidae, and the genus Sus. They can be used in the treatment of a variety of cancers, including liver cancer, lung cancer, breast cancer, and so on. Segatto et al. experimentally demonstrated that transgenic cancer pigs exhibit a high similarity to human cells when facing bladder cancer, making them a valuable research model for liver and lung cancer as well [74]. In the exploration of breast cancer pathogenesis, researchers discovered that the breast cancer susceptibility gene (BRCA1) gene in pigs encodes a nuclear protein consisting of 1863 amino acids, identical to that in humans. Donninger et al. pioneered the porcine breast cancer model and determined that the inactivation of the BRCA1 in porcine cells promotes phenotypic switching, validating the use of pigs as a model for studying BRCA1-defective breast cancer and establishing the first porcine breast cancer cell line [75].

#### 2.2.3. Scandentia (Mainly Tree Shrews)

The classification of the tree shrews’ status still remains a topic of debate. While some scholars categorize them as insectivorous, others place them within the primates, considering them to be one of the most primitive primate stems. In recent years, new insights have led to the classification of tree shrews as an independent class called Scandentia. Tree shrews, as experimental animals, possess the advantages of small size, rapid reproduction, and easy feeding due to their close relation to primates. Through whole-genome sequencing, it has been discovered that tree shrews share a closer evolutionary hierarchy and phylogenetic domain with humans [76]. A previous study by Zeng et al. has demonstrated the spontaneous development of breast cancers in tree shrews, leading to the generation of a tree shrew breast cancer model using lentivirus-expressing. Phosphatidylinositol-4,5-Bisphosphate 3-Kinase Catalytic Subunit Alpha (PIK3CA)-H1047R [77]. Additionally, a pancreatic cancer model has also been established using lentiviruses in recent years.

#### 2.2.4. Carnivores (Mainly Beagle Dogs and Cats) 

Among the carnivore orders, beagle dogs and cats are commonly employed as preclinical animal models. Beagle dogs are commonly used as the primary model for preclinical pharmacokinetic studies. The spontaneous occurrence of cancers in dogs closely resembles human cancers in terms of clinical presentation, histological features, molecular characteristics, response to and resistance to therapy, and the development of resistant metastases [78]. Prostate and breast cancers serve as the primary models, and the recent preclinical trials conducted by Massiere et al. have shown that beagle dogs can also be utilized in studies related to colorectal cancer (CRC), clear cell renal cell carcinoma (ccRCC), and PDAC [79]. The anatomical similarity between the prostate of dogs and humans has made it a valuable model for studying spontaneous prostate cancer [80]. Liu et al. demonstrated that SY-707 is a validated anaplasticlymphoma kinase/ focal adhesion kinase/insulin-like growth factor 1(ALK/FAK/IGF1R) inhibitor based on its preclinical efficacy in inhibiting growth and metastasis in a beagle model of breast cancer cells [81].

Additionally, cats have been utilized as a preclinical model for breast cancer due to its high similarity with human breast cancer in terms of age of onset, incidence, histopathological features, and metastatic patterns [82]. Over 80% of feline breast tumors are malignant and exhibit rapid development and early-stage metastasis, making them an ideal model for aggressive breast cancer. However, unlike other large animals, the cat preclinical model requires the inoculation of cultured tumor cells into mice for further analysis.

#### 2.2.5. Primates (Mainly Cynomolgus Monkeys)

Non-human primates, especially cynomolgus monkeys, are closely related to humans. They share the highest genetic homology with humans, ranging from 75% to 98.5%. Non-human primates have shown significantly superior results compared to other animal models in preclinical studies of biopolymer drug candidates. Simon et al. propose cynomolgus monkeys as a translational model for cancer immunotherapy, with examples including CRC and BRCA [83].

Development of Different Preclinical Animal Models 

Figure 1 depicts the evolution of preclinical animal model utilization over time. The first row displays various preclinical animal models, the second row presents the cancers for which the animal model is predominantly utilized, and the arrows below indicate the years when each animal model was initially employed in cancer treatment.

In the early 18th century, rats were first domesticated and subsequently utilized in animal experiments during the mid-19th century. Currently, rats play a crucial role in life science, medical research, and healthcare. They are the second most commonly used mammalian experimental animals after mice. Esophageal cancer is a prevalent and highly incident malignant tumor of the digestive tract. Rats’ anatomical and physiological similarities have been leveraged for studying esophageal cancer in its early stages. Moreover, rat models are employed in researching treatments for various cancers, such as breast cancer and acute myeloid leukemia [84,85]. Cats, known as domestic pets, have been utilized as experimental animals since the late 19th century. Sherrington’s stretch reflex experiment not only advanced people’s understanding of movement but also established cats as valuable experimental subjects across various fields of research. In oncology, early-stage cats are commonly used as representative models for aggressive breast cancer, and recently, preclinical cat models of head and neck squamous cell carcinoma have been under investigation [86,87]. Mice are widely recognized as the most commonly utilized preclinical experimental animals globally. The DBA strain of mice was first introduced in the 1910s, and various strains have been developed since then. CRC is among the most frequently diagnosed and severe cancers, ranking within the top three worldwide in terms of cancer statistics. The mouse model of colorectal cancer can replicate the occurrence and progression of human colorectal cancer, holding significant theoretical value and clinical importance in studying disease mechanisms, discovering new drug targets, and evaluating preclinical pharmacodynamics. It is now almost universally employed in studies involving various types of cancer tumors in mouse models, underscoring its significance. Beagle dogs have been utilized in animal experimental research since the 1950s due to their small size, gentle temperament, consistent response, high reproducibility, well-developed brain, and strong adaptability. They are extensively employed in biological and medical research, particularly in non-clinical drug studies. Prostate cancer is among the most prevalent malignant tumors in the genitourinary system of middle-aged and elderly men. Due to its anatomical similarity to humans and responsiveness to treatment, beagles have been widely used as a model for hormone-induced benign prostatic hyperplasia (BPH) research [88]. Beagle dogs have also been selectively bred for various tumor models, including thyroid cancer and breast cancer, and continue to play a pivotal role in toxicology experiments [89,90]. In 1959, Wostomalm successfully achieved the first breeding of sterile rabbits through cesarean section uterine extraction and artificial lactation. Various grades of experimental rabbits were established and widely utilized in scientific research and product quality verification. The Rabbit VX2 tumor model is a commonly used transplantation tumor model, and new research suggests that rabbits can also serve as a model to simulate the occurrence and development of osteosarcoma [91,92]. In the 1960s, the newly established Kunming Institute of Zoology founded a primate domestication and breeding center for scientific research, focusing on cynomolgus monkeys. Cynomolgus monkeys, as non-human primates closely related to humans, have been extensively utilized in various studies. Their application extends to pharmacokinetic research on bispecific antibodies in lymphoma and the development of antibody-drug conjugates (ADCs), thereby broadening their potential applications in fields such as breast cancer [93,94]. Due to the physiological and anatomical similarities, nutrient metabolism, biochemical indexes, and other characteristics shared between pigs and humans, particularly in the structure of the cardiovascular system, which closely resembles that of humans, pigs are considered ideal experimental animals. Miniature pigs were initially utilized in medical research in Europe before being introduced to the United States in the 1980s. An American Sinclair miniature pig strain was found to develop spontaneous cutaneous melanoma in 80% of the animals, making it a long-standing model for cutaneous melanoma [95]. Research by Niels et al. demonstrated that miniature pigs could also be valuable in studying prostate cancer and assessing growth distribution [96]. Tree shrews, being small mammals approximately the size of laboratory rats and close relatives of primates, are anticipated to supplant nonhuman primates in certain biomedical research and applications due to their short reproductive cycle, low feeding cost, and large single-litter size. Phylogenetically closer to humans and prone to spontaneous breast cancers, tree shrews hold significant potential for breast cancer research owing to their closer genetic relationship with humans. As mentioned earlier, the tree shrew breast cancer model has been utilized for gene editing therapy. The discovery of Lipid nanoparticles (LNPs) delivery system based on SM-102 could serve as a promising treatment strategy against hepatitis B virus (HBV) infection, effectively preventing liver cancer [97].

Now that there are so many preclinical experimental animals, how exactly are they useful for cancer treatment? How should we properly use preclinical experimental animals for testing? This will be detailed below.

## 3. Application of Preclinical Animal Models in Cancer Therapy

### 3.1. Study of the Characteristics of Different Stages of Tumor Development and Anti-Cancer Drug Development (Mainly Mice)

In the exploration of various stages of tumor development and anti-cancer drug development, with a specific focus on rodent models, it is crucial to emphasize the intricate interplay of the multiple genetic factors that drive tumor initiation and progression. Animal models, especially rodents, are indispensable tools for unraveling the complex mechanisms underlying human tumorigenesis, providing a comprehensive understanding of the disease. A groundbreaking review highlights the evolving comprehension of cancer hallmarks, which have recently been expanded to include emerging features such as phenotypic plasticity and cellular senescence, along with enabling features like epigenetic reprogramming and microbiome diversity [98]. These advancements reflect a deeper understanding of the biological landscape of tumors, guiding the strategic design of preclinical studies.

Mouse models have played a pivotal role in translating this knowledge into practical applications, particularly in cancer drug development. By mimicking human cancer biology, they have facilitated the discovery and validation of numerous innovative therapies. For example, drugs targeting specific molecular pathways—informed by tumor marker research that has evolved through three definitive eras since 2000—have been successfully tested in rodent models before advancing to clinical trials [98,99,100]. This iterative process emphasizes the importance of understanding the specific traits targeted within cancer biology. At present, mouse tumor models can be categorized into spontaneous tumor mouse models, induced tumor mouse models, genetically modified tumor models, xenograft tumor models, and homografted tumor models. Researchers select these models based on the specific research needs. Typically, the C57BL/6 and BALB/c strains are chosen for spontaneous tumor modeling due to their well-established genetic backgrounds. C57BL/6 mice are commonly used to simulate human genetic diseases and individual differences, while smaller BALB/c mice offer genetic purity and are also widely utilized. Induced mouse tumor modeling involves the use of chemical carcinogens, such as MNU (N-nitroso-N-methylurea) and DEN (diethylnitrosamine), to induce various tumors in mice, including gastric cancer and gastric cancer [101,102]. Genetically modified mouse tumor models have seen a surge in popularity with increasingly diverse types available. These include conditional knockout models for tumor suppressor genes like P53 and Phosphatase And Tensin Homolog (PTEN) using P53 Flox and PTEN Flox mice [103,104], and point mutation (or conditional point mutation) cancer models for oncogenes such as the Kras-LoxP-Stop-LoxP (LSL)-G12D model [105,106]. The selection of a xenograft tumor model should be based on different sources. This section will focus on the biological characteristics of different cancers combined with preclinical mouse models mentioned above to describe their specific contributions to cancer treatment progress and anti-cancer drug development.

#### 3.1.1. Sustaining Proliferative Signaling

The chronic proliferation ability is a fundamental characteristic of cancer cells, as they release their control over growth-promoting signals and become self-regulating masters [21]. H-Ras (HRas Proto-Oncogene) oncogenes activation reflects this characteristic, as demonstrated in Feng et al.’s study on CRC. They found that H-Ras activates the downstream signaling of epidermal growth factor receptor (EGFR) and significantly inhibits the efficacy of MRTX1133, a KRAS-G12D inhibitor. Their use of a xenograft mouse model to block the EGFR/wild-type RAS signal transduction axis has significant implications for the treatment of the KRAS mutant CRC, while also highlighting the ongoing development of EGFR inhibitors [107].

#### 3.1.2. Evading Growth Suppressors

In addition to promoting and sustaining the characteristic ability of positive growth stimulus signals, cancer cells must also evade the potent negative regulatory proliferation programs within the cell, many of which are dependent on the function of tumor suppressor genes. The functional experiments in mice have confirmed the significance of a typical tumor suppressor factor, the retinoblastoma (Rb) gene. Mario et al. investigated the impact of specific alterations in tumor suppressor factor (Rb) and oncogene (Ras) on nature kill (NK) cell-mediated cytotoxicity in glioblastoma cell lines injected into severe combined immunodeficiency (SCID) mice through Rb deficiency and mutation [108]. In recent years, cell cycle-dependent kinase inhibitors (CDKs) have been widely utilized for inhibiting tumor cell proliferation. Catherine et al. identified a novel CDK2 inhibitor, INX-315, and utilized patient-derived xenograft (PDX) and transgenic mouse models to demonstrate its efficacy in both Cyclin E1 (CCNE1)-amplified cancer and CDK4/6i-resistant breast cancer, thereby presenting a potential strategy for cancer cells to evade growth inhibition [109].

#### 3.1.3. Resisting Cell Death

The theory established over the past 20 years suggests that cell apoptosis acts as a natural barrier to cancer development [110]. Cancer cells have the ability to evade apoptosis and continue dividing under abnormal conditions, often due to common causes such as loss of p53 tumor suppressor genes. The inactivation of the p53 protein prevents cells from perceiving DNA damage that would normally trigger apoptosis. The upregulation of anti-apoptotic B-cell lymphoma-2 (Bcl-2) family members and inhibitor of apoptosis proteins, along with counteraction of the anti-apoptotic effect of Bcl-2 homology 3 (BH3)-only proteins, allows cancer cells to avoid cell death even when damaged, leading to tumor growth [111]. BH3 analogues, which mimic BH3 proteins, can enhance pro-apoptotic signals and promote intrinsic pathways leading to cell apoptosis. Sarah et al. used preclinical mouse and human models of invasive lymphoma to identify factors predicting drug resistance in patients receiving myeloid cell leukemia-1 (MCL-1)-targeted BH3-mimetic drugs. They also identified treatment methods capable of overcoming these types of drug resistance: using chemotherapy drugs separately or in combination with BH3 simulation therapy [112].

#### 3.1.4. Enabling Replicative Immortality

The accumulation of evidence suggests that telomeres located at the ends of chromosomes play a crucial role in achieving immortality in cancer cell proliferation [113]. The immortalization of mutated cells, which ultimately form tumors, is attributed to their ability to maintain a sufficient length of telomere DNA to avoid triggering aging or apoptosis. This is most commonly achieved through the upregulation of telomerase expression or recombination-based telomere maintenance mechanisms, which are less common. As a result, the development of telomerase inhibitors has sparked new hope for anti-cancer treatment. Janina’s team assessed the therapeutic effect of the telomerase inhibitor Imetelstat in a high-risk neuroblastoma cell xenograft mouse model. The findings indicated that this inhibitor can reduce telomerase activity by approximately 50% and improve the survival rate among mouse populations, thus demonstrating that targeting telomerase may be a viable treatment option for high-risk neuroblastoma patients [114].

#### 3.1.5. Inducing Angiogenesis

The formation of tumors, like normal tissues, necessitates the acquisition of nutrients and oxygen, as well as the capacity to eliminate metabolic waste and carbon dioxide. The angiogenesis process leads to a new vascular system that fulfills these requirements. Vascular endothelial growth factor (VEGF) A is a prototypical stimulator of angiogenesis. Both hypoxia and oncogene signaling can elevate the expression of the VEGF gene [115]. Similarly, in order to combat tumors, it is necessary to develop VEGF inhibitors as an alternative approach. This primary solution also serves as a focal point for numerous scientists conducting research. Huang’s research team discovered through preliminary analysis that the BicC family RNA-binding protein 1 (BICC1) gene was overexpressed in human pancreatic cancer, and subsequently utilized xenotransplanted tumor cells and mice to confirm that BICC1 plays a pivotal role in the VEGF-independent angiogenesis process in pancreatic cancer, resulting in resistance to VEGF inhibitors and offering novel therapeutic targets for pancreatic cancer patients [116].

#### 3.1.6. Activating Invasion and Metastasis

The transformation of normal cells into malignant tumors is characterized by local invasion and distant metastasis. Cancer cells undergo changes in their shape, attachment to other cells, and extracellular matrix, with the most significant change being the loss of E-cadherin. It has been demonstrated that an increase in E-cadherin expression acts as an antagonist of invasion and metastasis, while a decrease enhances these phenotypes [117]. The activation of the tumor cellular–mesenchymal to epithelial transition factor (c-MET) signaling pathway by cancer cells frequently promotes tumor formation, invasive growth, and metastasis. As a result, hepatocyte growth factor/cellular–mesenchymal to epithelial transition factor (HGF/c-MET) inhibitors have emerged as a potential treatment for non-small cell lung cancer. Manish et al. conducted preclinical studies using immunodeficient mice to evaluate the use of HGF receptor-neutralizing antibody targeting Ewing sarcoma tumors. They concluded that combining novel CAR-T cell therapy with the HGF receptor-neutralizing antibody Rilotumumab (AMG102) could enhance therapeutic efficacy, not only in Ewing sarcoma (EWS) but also in tumors with an abnormal activation of the HGF/c-MET pathway [118].

#### 3.1.7. Deregulating Cellular Energetics

The chronic and uncontrolled cell proliferation, which is the essence of tumor diseases, not only involves uncontrolled cell growth but also entails the corresponding regulation of energy metabolism to promote cell division. Taking glycolysis as an example, cancer cells can reprogram their glucose metabolism by limiting their energy production mainly to glycolysis, even in the presence of oxygen, leading to a state known as aerobic glycolysis. Both the aforementioned aerobic glycolysis state and the soon-to-be-described inhibitors of aerobic glycolysis appear to be an emerging concept. Scientists have identified that the glycolytic enzyme phosphofructose kinase-1 (PFK1) platelet-type plays a crucial role in this process. Huang et al. reported on a homolog of human carboxymethyl butenol esterase-like (CMBL) and utilized xenograft mouse models to observe changes in tumor development. The results indicate that CMBL inhibits colorectal cancer growth by suppressing glycolysis and provide potential combined strategies for treating CMBL-deficient CRC [119].

#### 3.1.8. Avoiding Immune Destruction

The immune surveillance theory proposes that the immune system constantly monitors cells and tissues, identifying and eliminating early cancer cells to prevent new tumors. The increased incidence of certain cancers in immunocompromised individuals supports the idea of immune system deficiencies in tumor monitoring [120]. Recent evidence from genetically engineered mice has demonstrated the crucial role of the immune system as a barrier against tumor formation and development. Given the nature of tumors, it is logical to focus on activating immunity, with the cytotoxic T lymphocyte-associated antigen-4 (CTLA-4) monoclonal antibody being a promising option. Bulion et al. tested the anti-tumor activity of plant-derived bispecific monoclonal antibodies targeting programmed cell death protein 1 (PD-L1) and CTLA-4 in vivo using humanized BALB/c mice carrying CT26 colorectal tumors, demonstrating a reduction in tumor volume and weight, which validates this treatment approach [121].

#### 3.1.9. Tumor-Promoting Inflammation

The inflammatory response can enhance various biomarker functions in the tumor microenvironment by releasing bioactive molecules, such as growth factors for sustaining proliferation signals, survival factors for inhibiting cell death, angiogenic factors, and extracellular matrix-modifying enzymes that facilitate angiogenesis, invasion, and metastasis. Additionally, it can also induce signals leading to epithelial–mesenchymal transition activation and other biomarker promotion programs. Unexpectedly, tumor-related inflammatory reactions may promote the initiation and progression of tumors [122]. Effective anti-inflammatory medications are being developed to significantly impede tumor development. Julia’s team has devised a treatment plan for hormone-refractory prostate cancer, evaluating the potential of immune proteasome inhibition in prostate cancer (PC) anti-inflammatory and direct anti-tumor therapy through observing its therapeutic effect on transgenic prostate adenocarcinoma mice. The results demonstrated that immunosuppressive proteasome exhibited significant therapeutic effects on PC progression in vivo and prevented tumor recurrence in castration-resistant prostate carcinoma-transgenic adenocarcinoma of the mouse prostate (CRPC-TRAMP) mice by blocking immunosuppressive inflammatory responses in the tumor microenvironment [123].

#### 3.1.10. Genome Instability and Mutation

In the process of acquiring the mutated genes necessary for coordinated tumor development, cancer cells typically elevate the mutation rate. This variability is achieved through increased sensitivity to mutagens, the disruption of one or several components of the genome maintenance mechanism, or a combination of both [124]. As an empowering feature, the response plan for this characteristic is constantly evolving. One prominent example is the emergence of poly ADP-ribose polymerase (PARP) inhibitors. PARP inhibitors (PARPi) have the potential to enhance the effectiveness of radiotherapy, alkylating agents, and platinum-based chemotherapy by inhibiting DNA damage repair in tumor cells and promoting apoptosis. Currently, research and development on PARP inhibitors are progressing rapidly, such as Olaparib. The use of PARP inhibitor combination therapy is also quite common. Zu et al. utilized a mouse model with metastatic ovarian cancer sharing similar genetic characteristics and found that, under PARPi treatment with depleted growth factor receptor-binding protein 2 (GRB2), tumor-bearing mice maintained a higher survival rate. Further research indicated that nuclear GRB2 protects DNA at the quiescent replication fork from meiotic recombination 11 (MRE11)-mediated degradation in the BRCA2 replication fork protection axis. This study suggests that GRB2 could be a potential therapeutic target and predictive biomarker for patients undergoing PARPi combined with immunotherapy [125].

Although Hanahan added four new biological characteristics in the review in 2022, this review was not included due to the fact that new drugs targeting these four characteristics have not been developed and are not reported enough.

The following table presents a simplified overview of the latest advancements in tumor hallmarks and their relationship to the development of targeted drugs, as depicted in Table 2.

### 3.2. Guiding and Development of Personalized Cancer Therapy Strategies (Synthetic Lethality)

In the pursuit of guiding and developing personalized cancer therapy strategies, the complexity of tumor ecosystems underscores the necessity for multifaceted approaches beyond synthetic lethality alone [126]. While synthetic lethality has gained significant traction due to its potential to selectively target cancer cells, it is just one among several precision medicine strategies benefiting from preclinical animal models.

Precision medicine strategies, as advocated by the National Institutes of Health (NIH)’s “Towards Precision Medicine” initiative since 2011, encompass a wide range of methodologies designed to tailor treatments based on a patient’s unique molecular profile [127]. Preclinical animal models play a crucial role in facilitating the exploration of these strategies, including immunotherapies, targeted therapies, and epigenetic modifiers.

Synthetic lethality stands out as a particularly promising strategy exemplified by successful applications, such as PARP inhibitors in BRCA-mutated cancers. Moreover, the use of synthetic lethality extends to exploring combinations such as WEE1 G2 checkpoint kinase (Wee1) and checkpoint kinase 1 (CHK1) inhibitors in neuroendocrine prostate cancer (NEPC), with preclinical mouse models showing effective tumor growth inhibition. Additionally, inhibitors of ataxia–telangiectasia-mutated gene (ATM), ataxia–Telangiectasia-mutated- and Rad3-related (ATR), and cyclin-Dependent kinase 12 (CDK12) are also being developed with the aid of preclinical models.

In summary, while synthetic lethality holds great promise in the personalized cancer therapy era, it is essential to recognize that it is part of a broader arsenal of precision medicine strategies [127,128]. Each approach benefits from insights garnered through preclinical animal models that serve as a cornerstone for translating novel concepts into clinically viable and efficacious treatments. In this section, we will focus on synthetic lethal therapy and primarily introduce several related inhibitors and their application in preclinical animal models for cancer treatment.

#### 3.2.1. PARP Inhibitors (PARPis)

PARP inhibitors (PARPis), exemplified by olaparib’s groundbreaking demonstration of anti-cancer activity in mice in 2009, have significantly revolutionized the field of cancer therapy by exploiting defects in DNA repair mechanisms [129]. This milestone paved the way for subsequent FDA approvals of PARPis, such as olaparib, rucaparib, niraparib, and talazoparib, highlighting the successful translation of preclinical findings into clinical practice.

Ongoing preclinical research continues to enhance our understanding of PARPis’ potential, particularly in challenging contexts like pancreatic ductal adenocarcinoma (PDAC), where despite surgical intervention, recurrence rates remain alarmingly high [130]. Notably, Zhang and colleagues utilized mouse models harboring PDAC xenografts to investigate the combined therapeutic impact of a recombinant measles virus (rMV-Hu191) and olaparib on PDAC with intact BRCA1/2 genes. Their study not only revealed the inhibition of tumor growth but also extended survival in mice, validating a synthetic lethality approach. This synergistic effect underscores the value of PARPis in conjunction with other therapies targeting cancers with specific DNA repair deficiencies [131].

In essence, PARP inhibitors, derived from extensive research on DNA repair pathways, epitomize the power of precision medicine. By focusing on their collective impact across various cancers and emphasizing their role in synthetic lethality strategies, we can streamline our discussion while acknowledging the breadth of their applications and the wealth of existing research.

#### 3.2.2. Protein Arginine Methyltransferase 5 (PRMT) Inhibitors

PRMT can methylate a variety of proteins and play crucial roles in biological processes, such as gene expression, splicing, and DNA damage repair [132]. Among them, PRMT5 has garnered the most attention and is considered a potential oncogene [133]. The synthetic lethal effect of PRMT5/ Methylthioadenosine Phosphorylase (MTAP) inhibitors in tumors had been reported in 2016 [134]. The synthetic lethality component of PRMT5 involves MTAP, a tumor suppressor gene frequently deleted in tumors. Therefore, PRMT5/MTAP inhibitors are being considered as potential antitumor agents. Currently, several PRMT5 inhibitors have entered clinical trials, with GSK3326595 advancing to phase II [135]. 

Novel PRMT inhibitors are also under investigation in preclinical trials. Djajawi et al. generated genetically engineered mouse models comparing wild-type and CRISPR knockout B16 cells treated with programmed death 1 (PD1) targeting PRMT1. It was found that PRMT1 inhibits interferon-γ (Ifn γ)-induced major histocompatibility complex-I (MHC-I) expression, thereby suppressing CD8 T cell-mediated killing. The results indicate that PRMT1 KO tumors exhibit a modest growth disadvantage compared to wild-type tumors and further demonstrate that targeting PRMT1 can enhance immunotherapy for MHC-I low-expression tumors to achieve synthetic lethal effects [136,137].

#### 3.2.3. Wee1 Inhibitors

The Wee1 kinase was initially discovered in yeast and is a crucial member of the serine/threonine protein kinase family. Tumor protein p53 (TP53), which forms a synthetic lethal effect with Wee1, belongs to the tumor suppressor gene due to its association with various malignant tumors. The prevalence of TP53 mutations in cancer has led to extensive research on Wee1 inhibitors by global pharmaceutical companies, with ZN-c3 being the most advanced and reaching clinical stage II. This oral Wee1 inhibitor has shown promise in treating advanced solid tumors by inducing premature mitosis and causing cancer cell apoptosis through DNA damage [138]. 

In preclinical studies for neuroendocrine prostate cancer (NEPC), Nest et al. found an increased expression of WEE1 and CHK1 in NEPC cell lines, leading them to explore the potential treatment using a synthetic lethal combination of Wee1 and other inhibitors. Their experiments using NEPC transgenic mouse models demonstrated that treatment with the Wee1 inhibitor AZD1775 and CHK1 inhibitor SRA737 effectively inhibited tumor growth, providing a promising approach for NEPC treatment [139]. 

Furthermore, significant progress has been made in the research and development of ATM, ATR, CDK12, and other inhibitors this year, many of which have entered clinical or preclinical studies. Synthetic lethal therapy offers strong specificity and low side effects, making it an emerging trend in modern cancer drug research and development. With new experimental methods and screening techniques evolving rapidly, utilizing synthetic lethality for personalized treatment plans based on individual patient backgrounds will bring profound innovation to targeted therapy for cancer patients.

### 3.3. Selection of Safety Evaluation Experiments

Due to the unique risks associated with preclinical research, the review process for preclinical studies is highly rigorous. When selecting animal models for non-human experiments, they must undergo multiple safety evaluation tests. While conducting safety evaluation experiments, particularly toxicology experiments, a comprehensive consideration of the following four aspects is essential: ethical considerations, experimental conditions, animal welfare, and legislative requirements. Ethical consideration is the primary principle of conducting safe evaluation experiments. Researchers must strictly follow the ethical welfare of animals, 3R principles, and relevant regulations on experimental animals and respect experimental animals and scientific research. The ethics of experimental animals stipulates a set of strict animal care regulations: adequate comfortable living and eating conditions should be given during breeding, scientific researchers should not abuse animals, and euthanasia methods must be used to kill animals. Experimental conditions are important indicators of reasonable conduct, and toxicology experiments follow the principle of randomization, control, and repetition. Providing the appropriate experimental conditions is key to ensure the reliability of the experimental results. Laboratory animals should be kept under constant temperature, humidity, and light conditions, and a suitable space and comfortable living environment should be provided. Monitoring and maintaining the health of the animals, including the provision of proper diet and water, is also required. Animal welfare and legislative requirements are key means to protect animal rights and interests, and the experimental design must meet ethical requirements, that is, to protect animal welfare and rights and minimize pain and suffering to animals. Local and international animal protection laws and regulations must be followed during the experimental process, and necessary ethical reviews must be carried out. Some of the details are discussed in this section. 

#### 3.3.1. Acute Toxicity Test

The acute toxicity test is the initial stage of toxicity research, involving the testing of single or multiple exposures within a 24-hour period. It requires the use of both rodent and non-rodent species. The oral, inhalation, or transdermal route of exposure is commonly employed in mice or rats. The main focus is on determining the median lethal concentration (LD50), where a higher LD50 indicates lower toxicity and a lower LD50 indicates a higher toxicity [140]. The fixed-dose method is commonly employed for preclinical acute toxicity testing in rodents, with rats being the primary research subjects. Non-rodents are typically assessed using the approximate lethal dose method, and Beagle dogs and monkeys are considered suitable candidates. The development of preclinical new drugs relies heavily on acute toxicity testing. Nistuzumab, a therapeutic monoclonal antibody targeting EGFR, has been widely used as a therapeutic drug in numerous countries. Wendy’s team prepared the IRDye800CW nimotuzumab reagent for clinical trial applications and conducted a single-dose toxicity study using a mouse cancer model. The results indicated that the formulation was determined to be non-toxic from both acute and delayed toxicity studies, confirming that Nistuzumab coupled with IRDye800CW is safe and does not exhibit typical EGFR-targeted antibody-associated toxicities [141]. In the study of acute toxicity testing for compounds, the selection of experimental animals follows certain principles: Animals chosen should exhibit toxicity reactions similar to those in humans; they should be easy to feed, manage, and handle; be readily available and cost-effective. To better predict compound hazards in humans, it is necessary to select at least two experimental animals, preferably one rodent and one non-rodent, for calculating their acute toxicity parameters. Mammals are given priority in species selection. Rats and mice are commonly used in practice, with rats being particularly prevalent. It is important to note that rats are not always the most sensitive to foreign compounds. Rabbits are often employed for studying skin toxicity and mucous membrane irritation caused by compounds. Cats and dogs may also be used for acute toxicity tests, but their use is limited due to cost considerations. Pigs, as omnivores whose biological responses to some compounds resemble those of humans, especially regarding skin structure, are occasionally utilized despite their large size and higher cost [142,143,144,145,146,147]. In acute toxicity testing, the following methods are commonly utilized and acknowledged: sub-lethal dose, maximum tolerated dose, fixed-dose procedure, stepwise method, pyramid design, etc. Nevertheless, regardless of the method employed, adherence to fundamental laws and regulations as well as ethical considerations is imperative; legislative requirements are delineated in the literature [148,149].

#### 3.3.2. Subchronic Toxicity Test

The term “subchronic toxicity” refers to the adverse effects that occur in experimental animals after exposure to high doses of experimental drugs for multiple consecutive days, typically at doses lower than the acute lethal dose (LD50). The main purpose of subchronic toxicity testing is to observe toxic reactions, toxic doses, and pathological changes in target organs. This type of testing involves continuous administration for 4–13 weeks using mice, rats, rabbits, and dogs as test subjects. Subchronic toxicity test is the toxicity test in which the animal ingests the subject matter daily or repeatedly for about 1/10 of the life of the experimental animal. Regarding the specific duration, the duration is at least 3 months (2 to 6 months) for rodents and 1 year for dogs. Due to the prolonged duration of these tests, animals with relatively small body weights (or young age) are usually selected—for example, around 15g for mice and 100 g for rats. Additionally, non-human primates, such as Cynomolgus monkeys and tamarins, have also been included in subchronic toxicity experiments expanding the range of animal subjects [150]. The review report emphasizes that there is already a substantial amount of data on the toxicity and toxicokinetics of test substances in repeated dose toxicity studies with (sub)chronic duration, which can be used to reliably determine tolerable dose levels [151,152,153,154,155,156]. The design principles for the subchronic toxicity test are as follows: firstly, endeavor to replicate the route or manner in which humans come into contact with the compound in their environment; and secondly, align the exposure pathway expected for conducting chronic toxicity tests. The specific routes of contact mainly include three types: oral, respiratory, and cutaneous. Legislative requirements for subchronic toxicity experiments are described in the literature [157,158].

#### 3.3.3. Chronic Toxicity Test

The chronic toxicity test involves the long-term administration of low-dose compounds to experimental animals in order to observe their toxic effects. This test is conducted to determine the minimum dose and no observable adverse effect level of a drug, which indicates the potential harm caused by prolonged exposure to the compound [159]. It provides a toxicological basis for drug safety evaluation and human safety limit standards. Polyphenols have been demonstrated to possess anti-tumor efficacy. Ricardo et al. conducted a study on polyphenol extracts from herbs and validated their toxicity through a 28-day oral chronic toxicity assessment experiment using Wistar rats and New Zealand rabbits [151]. The purpose of chronic toxicity testing is to observe potential harm to the body resulting from the long-term ingestion of the tested substance and to determine both the maximum dose with no observed adverse effect (NOAEL) and the minimum observed adverse effect level (LOAEL) of the substance. This information can then be used to establish the maximum allowable amount of the substance in food as well as the acceptable daily intake (ADI) for humans. The NOAEL represents the highest dose (mg/kg body weight) at which animals show no toxic effects when exposed to a substance over a specified period, typically spanning most or all of their lifespan. On the other hand, LOAEL refers to the lowest dose (mg/kg body weight) that causes minimal toxicity within a defined timeframe, also known as the threshold dose. Preclinical animal toxicity experiments can provide valuable references for the application of various drugs in humans. The recommended experimental periods vary for different animals, with rodents typically tested for a period spanning from 6 months to 2 years, while mammals require longer periods, such as 1.5 years for mice, 2 years for rats, and 6 years for beagles according to the World Health Organization [160,161,162,163,164,165]. The content and methodologies of subchronic and chronic toxicity tests are essentially similar, with the primary distinction lying in the duration of observation. Applicable regulations mandate that these two types of tests can be integrated and compared.

#### 3.3.4. Reproductive Toxicity Test

Reproductive toxicity experiments involve assessing the impact of drugs on the reproductive function and developmental processes of mammals, particularly rodents, in order to predict their potential effects on parental reproductive functions, such as germ cells, conception, pregnancy, childbirth, and lactation, as well as their effects on embryo development and postnatal growth. These tests typically follow a three-stage design comprising general reproductive toxicity tests, teratogenic sensitivity tests, and perinatal tests. Different animal species exhibit varying sensitivities to drugs; therefore, at least two or more animals should be used for testing. Rats are commonly chosen for these experiments due to their practicality, high comparability with other experimental results, and extensive background information available. Non-rodents are usually represented by rabbits due to their accessibility and practicality; however, some studies may also use beagles or monkeys. The ultimate objective of animal reproductive toxicity testing is to anticipate potential reproductive and developmental toxic reactions in humans [166,167,168,169,170,171].

#### 3.3.5. Drug Dependence Test

The term “drug dependence” refers to the necessity for the repeated administration of medication in the body due to the pharmacological effects of drugs on physiology or the mind, either to induce a sense of well-being or to alleviate discomfort. The observation period is generally prolonged and encompasses various types of studies. Suitable experimental animals include rodents (rats and mice), dogs, and primates. Both the International Council for Harmonisation of Technical Requirements for Pharmaceuticals for Human Use (ICH) M3 document and the European Medicines Agency (EMA) 2006 guideline indicate that rodents are the preferred species for assessing drug dependence, unless there are specific requirements for primate use. Interestingly, there are still discrepancies in animal selection among different drug regulatory agencies; in Asia, primates tend to be favored, while in Europe, it is rats [172,173,174,175,176].

#### 3.3.6. Carcinogenicity Test

The purpose of carcinogenic experiments is to observe the potential carcinogenic effects of drugs in animals, with the aim of evaluating and predicting their potential harm to the human body. Research on drug carcinogenicity typically focuses on rats or mice as experimental subjects, involving the continuous daily administration and observation of tumor incidence rates in test animals over a 2-year period. It should be noted that carcinogenic testing requires stringent criteria, including: (1) high standards for animal selection, commonly using F344 rats, A-line mice, gene knockout mice, etc.; (2) strict environmental requirements encompassing physical, chemical, and biological carcinogenic factors; and (3) the exclusion of all other potential carcinogens [177,178].

#### 3.3.7. Genotoxicity Test

Genetic toxicity research involves the assessment of drug effects on mutations and chromosomal damage in organisms such as bacteria and cells. Various methods for genetic toxicity testing exist, including in vitro and in vivo tests. Currently accepted animal tests for genetic toxicity include bone marrow micronucleus testing and mammalian chromosome aberration testing [179,180], with mice and rats being commonly used experimental animals [181,182,183,184,185].

The aforementioned preclinical animal models are recommended to varying extents for different safety evaluation experiments, as outlined in Table 3.

**Figure 3:**
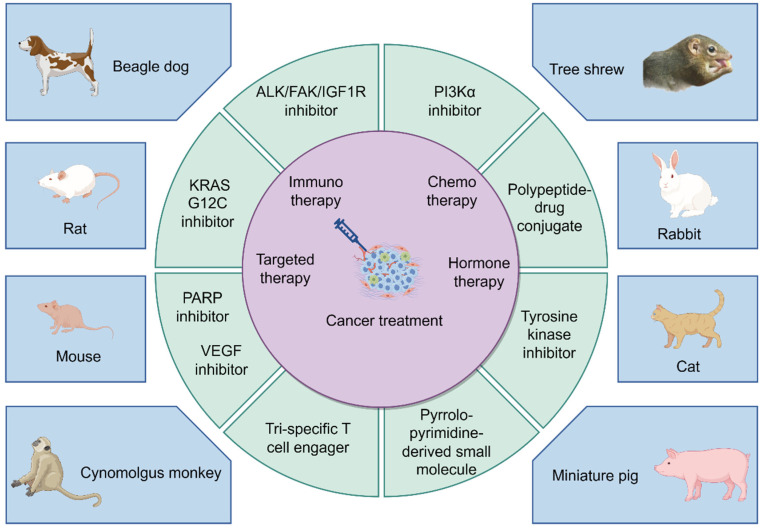
A schematic diagram illustrating the interaction between cancer treatment and preclinical animal models (By FigDraw).

While Figure 3 provides a schematic diagram illustrating the interaction between cancer treatment and preclinical animal models. Rodents (rats and mice) are still widely used due to their highly homologous genome and physiological function construction with the human body, as well as their ability to simulate the human environment well during spontaneous tumor occurrence and development [186]. Dogs, especially beagles, are undoubtedly the preferred choice among experimental animals due to reliable biological characteristics, convenient administration methods, examination procedures, and blood collection without anesthesia requirement. Primates, represented by Cynomolgus monkeys, also hold important application prospects. Each of the new drugs produced in preclinical animals shown in the figure cites the following literature (Mouse: PARP inhibitor, VEGF inhibitor [116,117,118,119,120,121,122,123,124,125], Rat: KRAS G12C inhibitor [187,188,189], Beagle dog: ALK/FAK/IGF1R inhibitor [81], Cynomolgus monkey: Tri-specific T-cell engager [190], Tree shrew: PI3Kα inhibitor [77], Rabbit: Polypeptide drug conjugate [191], Cat: Tyrosine kinase inhibitor [192,193], Miniature pig: Pyrrolo-pyrimidine-derived small molecule [194]). Future advances in science and technology will highlight the strengths of other large animals contributing to preclinical research.

## 4. Prospects and Challenges of Preclinical Animal Models

In recent years, preclinical animal models have made significant contributions to fields such as toxicology research, drug development, vaccine research, genetics, and neuroscience. With the advancement of biomedical technology, the application range of animal models has expanded, offering new possibilities for solving clinical problems. 

However, it is challenging for any animal model to fully replicate the highly intricate biological systems within the human body. Despite extensive efforts invested by researchers in preclinical animal models, disparities in physiology, pathology, and other aspects between animals and humans can result in the incomplete translation of research findings. Moreover, the establishment of animal models entails long cycles and high costs, limiting their widespread application and promotion. Ethical concerns surrounding animal experimentation also exist. Researchers should prioritize the welfare of animals, minimize their stress and suffering, respect their lives, refrain from cruel treatment, and employ the least distressing methods when handling them. This aligns with the internationally advocated 3R principle—Reduction, Replacement, and Refinement. 

The “3R principles,” initially proposed by Russell and Burch in 1959, encompass guidelines for conducting laboratory animal experiments to safeguard the welfare of experimental animals and ensure the scientific integrity of data obtained from these experiments. These principles emphasize the replacement and refinement of and reduction in experimental animal usage. Further details are outlined below:

Reduction: Minimize the usage of laboratory animals in experimentation while maintaining data quality and accuracy. Strive to minimize their involvement unless necessary for explaining experiment outcomes. Effective strategies include the judicious selection of subjects for experimentation; meticulous study design; and efficient utilization and appropriate modes for experimentation.

Replacement: Endeavor to avoid using live subjects whenever possible by employing alternative methodologies with equivalent objectives such as lower organisms over higher ones; smaller species rather than larger ones; histological studies replacing whole-animal tests; molecular biology techniques substituting traditional animal-based approaches; synthetic materials supplanting live subject trials; and computational modeling simulating physiological responses without live subjects.

Refinement: Mitigate harm inflicted on research subjects through humane treatment measures that encompass enhancing living conditions and care protocols for laboratory specimens; and refining specimen selection criteria along with technical procedures and methodological optimization during experimentation processes to minimize physical distress or suffering experienced by research subjects, thereby ensuring scientifically valid outcomes.

Experimental animal ethics encompasses the social moral standards and principles guiding the human treatment of experimental animals and conduct of animal experiments. The “3R” principle serves as the cornerstone of experimental animal ethics and a key criterion in the ethical review of animal experiments. Researchers are expected to cultivate an awareness of animal welfare and ensure the protection of animals involved in experiments. Scientific evaluation should be employed to assess the pain and distress experienced by animals, with timely consideration given to humane endpoints. When euthanasia is necessary, it should be carried out in a manner that minimizes or eliminates panic and suffering, allowing for quiet and swift passing.

In the future, as the preclinical animal model system becomes more advanced, researchers can further enhance their technical capabilities. For instance, they can focus on improving and optimizing animal models to better replicate human physiology and disease states, thereby enhancing the accuracy and reliability of research. Additionally, they can explore ways to better uphold and safeguard the rights and welfare of animals while advancing scientific research. Although there is still a long road ahead, we have reason to believe that the emergence of interdisciplinary approaches and new technologies will inevitably lead to the development of more advanced animal models, ultimately benefiting a large number of patients.

## Figures and Tables

**Table 1:** Summary of the advantages and disadvantages of the five mouse models and the latest progress in specific cancers.

Model Type	Areas of Application	Advantages	Disadvantages	Recent Advances in the Treatment of Specific Cancers
Transplantation model (homologous, xenogeneic)	Evaluate tumor immunotherapy in tumor-bearing mice	Homologous: Origin is consistent. Xenogeneic: A variety of animal models.	Homologous: not universal, immune difference, and results change frequently. Xenogeneic: limitations of species differences, clinical application of low success rate, and key differences between species.	Homology: AMG509 mouse model—metastatic castration-resistant prostate cancer [47]. Xenogeneic: Mouse models of NSCLC—aerobic lung cancer [48].
In situ model	Evaluate growth in the appropriate microenvironment/ local tumor invasion/ preclinical survival end points	Chemical carcinogen induction method: commonly used DMBA, AOM, etc. Gene-editing technology, such as CRISPER.	Chemical carcinogen induction: toxic to normal cells. Gene-editing technology: high cost, complex methods, and low success rate.	Chemical carcinogen induction: AOM-DSS mouse model—colorectal cancer [57]. Gene editing technology: CRISPR-Cas9-mediated somatic glioblastoma (GBM) mouse models [58].
Genetic engineering mouse model	Identify potential tumor genes and drug targets, analyze the effects of the tumor microenvironment, and evaluate drug resistance mechanisms	Gene knockout mice: evaluate the function of oncogenes and tumor suppressor genes. Humanized mice: a real simulated microenvironment.	Model is expensive, the production cycle is long, and the single model lacks representativeness.	Gene knockout mice: Ucp2 KO mouse model—pancreatic ductal adenocarcinoma (PDAC) [60]. Humanized mice: The HIS (humanized immune system) mouse model for CAR-T therapy [61].
Primary tumor xenograft model	Clinical relevance and predictive value, drug screening and development, and tumor biology research	Preserving the genetic and epigenetic diversity of the tumor in vivo.	Cannot be repeatedly obtained, long time, and unstable success rate.	NOD-SCID-IL2RgammaC-null (NSG) mice—lung cancer [65]. PDX mouse models of highly invasive (HI) and minimally invasive brain metastases (MI BrM) [66].
Chemical carcinogenesis model	Developmental sequelae that are molecularly, biochemically, and histopathologically similar to specific human cancers	Simple operation, low time cost, and play interventions before cancer occurs.	Mechanism has not been elucidated; toxicity of carcinogens; a gap between experimental differentiated tumors and human tumors.	Diethylnitrosamine (DEN)—hepatocarcinogenesis [70]. Benzo(a)pyrene—lung cancer [67].

**Table 2:** Cancer hallmarks, new drugs targeting cancer hallmarks, and the latest progress in research and development using mouse models.

Cancer Hallmarks	New Drugs	Mouse Model—Cancer—Drug/Protocol Name
Sustaining Proliferative Signaling	Epidermal growth factor receptor inhibitor	Xenotransplantation mice—KRAS mutation CRC—MRTX1133 [107]
Evading Growth Suppressors	Cell cycle-dependent kinase inhibitor	Transgenic mice—breast cancer—INX-315 [109]
Resisting Cell Death	BH3 analogue	Homologous transplantation mice—lymphoma—Vnetoclax [112]
Enabling Replicative Immortality	Telomerase inhibitor	Xenotransplantation mice—High-risk neuroblastoma—Imetelstat [114]
Inducing Angiogenesis	VEGF inhibitor	Xenotransplantation mice—pancreatic cancer—Gmcitabine [116]
Activating Invasion and Metastasis	HGF/c-MET inhibitor	Homologous transplantation mice—Ewing sarcoma tumors—CAR-T therapy+AMG102 [118]
Deregulating Cellular Energetics	Aerobic glycolytic inhibitor	Xenotransplantation mice—colorectal cancer—CMBL [119]
Avoiding Immune Destruction	CTLA-4 monoclonal antibody	Xenotransplantation mice—colorectal cancer—Dual variable domain immunoglobulin atezolizumab × 2C8 [121]
Tumor-promoting Inflammation	Anti-inflammatory drug	Transgenic mice—prostate cancer—Immunoproteasome [123]
Genome Instability and Mutation	PARP inhibitor	Homologous transplantation mice—ovarian cancer—GRB2+(Olaparib/Talazoparib) [125]

**Table 3:** Relationship between safety evaluation experiments and the selection of preclinical animal models.

	Test Types	Acute Toxicity Test		Subchronic Toxicity TEST		Chronic toxicity Test		Reproductive Toxicity Test		Drug Dependence Test		Carcinogenicity Test		Genotoxicity Test	
Animal Species															
Mouse		✓✓	[144]	✓✓	[152]	✓✓	[161]	✓	[167]	✓✓✓	[172]	✓✓✓	[177]	✓✓✓	[184]
Rat		✓✓✓	[143]	✓✓✓	[155]	✓✓✓	[164]	✓✓✓	[166]	✓✓✓	[175]	✓✓✓	[178]	✓✓✓	[182]
Beagle dog		✓✓✓	[142]	✓✓✓	[151]	✓✓✓	[163]	✓✓	[171]	✓✓	[174]	NR		✓	[181]
Rabbit		✓	[147]	✓✓✓	[153]	✓	[160]	✓✓✓	[170]	✓	[176]	NR		✓	[185]
Cynomolgus monkey		✓✓✓	[146]	✓✓✓	[154]	✓✓✓	[165]	✓✓	[168]	✓✓✓	[173]	NR		✓	[183]
Miniature pig		✓	[145]	✓	[156]	✓✓✓	[162]	✓✓	[169]	NR		NR		NR	

Note: ✓✓✓ indicates highly recommended; ✓✓ indicates recommended; ✓ indicates not recommended; NR indicates not reported.

## Data Availability

All datasets generated for this study are included in the article.
